# Glandular odontogenic cyst: a ten-case series and literature review with emphasis on radiological diagnostic challenges

**DOI:** 10.1186/s12903-025-07296-1

**Published:** 2025-11-26

**Authors:** Young-Eun Kwon, So-Young Choi, Mee-seon Kim, Chang-Hyeon An, Seo-Young An

**Affiliations:** 1 Department of Oral and Maxillofacial Radiology, Kyungpook National University School of Dentistry, IHBR, Daegu, Republic of Korea; 2 Department of Oral and Maxillofacial Surgery, Kyungpook National University School of Dentistry, Daegu, Republic of Korea; 3 Department of pathology, Kyungpook National University School of Dentistry, Kyungpook National University Hospital, Daegu, Republic of Korea; 4 Department of Oral and Maxillofacial Radiology, Kyungpook National University School of Dentistry, IHBR, ITRD, Daegu, Republic of Korea

**Keywords:** Odontogenic cysts, Odontogenic tumors, Diagnosis, differential, Radiography, panoramic, Cone-Beam computed tomography

## Abstract

**Background:**

Glandular odontogenic cyst (GOC) is a rare developmental odontogenic lesion characterized by histological complexity and significant radiological overlap with other cystic or neoplastic jaw lesions. Its nonspecific imaging features often lead to misdiagnosis, delaying appropriate treatment and increasing the risk of recurrence. Despite its clinical relevance, radiographic analysis of GOC remains underreported in the literature.

**Methods:**

This retrospective study analyzed ten cases of histopathologically confirmed GOC diagnosed between 2015 and 2024 at Kyungpook National University Dental Hospital. All patients underwent both panoramic radiography and cone-beam computed tomography (CBCT) for preoperative imaging evaluation. Radiographic features were assessed and compared with final histopathological diagnoses. Additionally, a comprehensive literature review was performed using PubMed. A total of 74 previously reported cases were included based on the availability of both imaging and histological data. Descriptive statistical analysis was performed to summarize demographic data, radiographic patterns, and frequency of preoperative misdiagnoses.

**Results:**

Ten histopathologically confirmed GOC cases demonstrated male predominance (7:3), a mean age of 49.1 years, and frequent mandibular involvement (90%). Most lesions were unilocular with variably defined margins and were commonly misdiagnosed as odontogenic keratocyst (OKC) or radicular cyst (RC). One case showed recurrence after 55 months. In the literature, 74 reported GOC cases had a mean age of 44.4 years, with similar male predominance and mandibular predilection (75%). Radiographically, 51.7% were multilocular, 93.2% had well-defined margins, and 14.6% demonstrated recurrence. A literature review confirmed that OKC and ameloblastoma (AB) were the most frequently misdiagnosed entities.

**Conclusion:**

GOC can exhibit radiographic overlap with a wide range of odontogenic lesions, including OKC, RC, and AB, reflecting its imaging variability and preoperative diagnostic challenges.

## Background

Glandular Odontogenic Cyst (GOC) is a rare developmental odontogenic cyst that originates from remnants of the dental lamina with glandular differentiation [1]. It was first described as a “sialo-odontogenic cyst” in 1987 by Padayachee and Van Wyk, who noted its histological resemblance to salivary gland structures [2]. However, Gardner et al. later introduced the term GOC in 1988, emphasizing its unique glandular-like histopathological characteristics [3]. In 1992, the World Health Organization (WHO) classified it as a distinct odontogenic cyst due to its characteristic epithelial features, including glandular or duct-like structures, intraepithelial microcysts, and mucous cells, which differentiate it from other odontogenic cysts [4]. The 2022 WHO classification of GOC highlights the necessity of diagnosis based on multiple histopathological features rather than a single criterion, with hobnail cells being the most characteristic marker [5].

Despite its classification, GOC remains one of the most diagnostically challenging odontogenic cysts, primarily due to its radiological overlap with other cystic lesions such as lateral periodontal cysts, botryoid odontogenic cyst (BOC), and even low-grade central mucoepidermoid carcinoma (MEC) [6]. Radiographically, GOC presents as a well-defined radiolucent lesion, which may appear as either unilocular or multilocular, often exhibiting scalloped borders and cortical expansion [7].​ Larger lesions tend to be multilocular, often leading to misdiagnosis as an odontogenic keratocyst (OKC) or ameloblastoma (AB) [8]. Additional radiographic findings such as root resorption and tooth displacement further complicate the differentiation from other odontogenic pathologies [8].

Histopathologically, GOC is characterized by an epithelial lining composed of cuboidal or columnar cells, mucous-producing cells, intraepithelial microcysts, and ciliated epithelium, which contribute to its resemblance to central MEC, necessitating careful histopathological evaluation for accurate diagnosis [1, 9]. The presence of variable epithelial thickness, intraepithelial microcysts, and mucous cells further differentiates GOC from other similar lesions, such as intraosseous MEC [6]. Notably, MEC can be distinguished from GOC by the presence of MAML2 rearrangement, which is typically absent in GOC [8].

The reported prevalence of GOC is low, accounting for less than 0.5% of all odontogenic cysts, with a slight male predilection and a mean age of presentation around 50 years [10]. It predominantly occurs in the mandible, particularly in the anterior region (46%), though cases in the maxilla have also been documented [1]. Despite its benign nature, GOC exhibits aggressive growth potential and a high recurrence rate, ranging from 21% to 55%, particularly following conservative surgical treatment [8]. While enucleation is the most commonly performed treatment, it is often associated with high recurrence rates, necessitating more aggressive approaches such as peripheral ostectomy or marginal resection in larger or multilocular cases [8]​.

GOC is a rare but aggressive odontogenic cyst characterized by a high recurrence rate and a complex histopathological features [8, 10]. Diagnosing GOC preoperatively based on imaging alone is extremely challenging due to its nonspecific radiological features, often leading to misdiagnosis as other odontogenic cysts or benign tumors. Our institution has collected ten cases of GOC over the past decade. Through case analysis, this study aims to explore the wide spectrum of differential diagnoses that GOC can be confused with, emphasizing the radiological diagnostic challenges in order to enhance its accurate identification. In addition, by integrating our institutional cases with a comprehensive literature review, this study also aims to refine the understanding of GOC’s radiological presentation and improve differential diagnosis strategies for clinicians and radiologists.

## Methods

### Ten-Case series of GOC

This retrospective study analyzed ten cases of GOC diagnosed at the Department of Oral and Maxillofacial Surgery, Kyungpook National University Dental Hospital (KNUDH) between 2015 and 2024. Due to its retrospective nature, the Kyungpook National University Dental Hospital Institutional Review Board (KNUDH IRB) granted an exemption from informed consent (IRB number: KNUDH-2025-01-04-00).

Only cases with a histopathologically confirmed diagnosis of GOC following surgical excision were included. Additionally, patients who underwent both panoramic radiography and cone-beam computed tomography (CBCT) as part of their preoperative imaging assessment were selected, as these imaging modalities provided essential data for radiographic evaluation. Cases exhibiting radiographic features overlapping with other odontogenic cystic lesions and requiring differential diagnosis were prioritized for inclusion. Radiographic images were reviewed by three board-certified oral and maxillofacial radiologists, and discrepancies were resolved by consensus. Patients with incomplete imaging data or inadequate histopathological records were excluded from the study. Demographic information, clinical presentation, radiographic findings, and treatment approaches were analyzed for all included cases.

### Literature review

A comprehensive literature review was conducted using PubMed database to gather case reports, retrospective studies, and clinical analyses focusing on the radiographic characteristics of GOC and its differential diagnoses. The search strategy incorporated Medical Subject Headings (MeSH) and relevant keywords to ensure a targeted and exhaustive review of imaging-based studies. From the selected studies, data on patient demographics, lesion location, radiographic features (e.g., radiolucency, locularity, border definition, cortical bone involvement), and preoperative diagnostic impressions were extracted. Descriptive statistics—including frequencies, percentages, means, and standard deviations—were used to analyze trends in patient demographics, anatomical site involvement, radiographic characteristics, and the distribution of preoperative diagnostic interpretations in the literature.

### Search strategy

A comprehensive literature search was conducted in PubMed, with the final search completed in December 2024. The search strategy was designed to identify relevant studies on the radiographic features, differential diagnosis, and comparative imaging analysis of GOC. The search queries were structured into four primary categories. First, studies on radiographic features and imaging modalities were identified using terms such as *“Glandular Odontogenic Cyst”* combined with imaging techniques including *CBCT*,* computed tomography (CT)*,* panoramic radiography*,* multilocular radiolucency*,* unilocular radiolucency*,* and magnetic resonance imaging (MRI).* Second, to explore differential diagnosis, searches included *“Glandular Odontogenic Cyst”* in conjunction with *odontogenic keratocyst*,* ameloblastoma*,* dentigerous cyst*,* radicular cyst*,* and central mucoepidermoid carcinoma*. Third, for comparative analysis with other odontogenic cysts and tumors, the query *“GOC” AND (“Odontogenic Tumors” OR “Odontogenic Cysts”) AND (“Radiographic Features” OR “Imaging”)* was used. Lastly, case reports and clinical findings were retrieved using *“Glandular Odontogenic Cyst” AND “Case Report”* to examine individual case presentations and diagnostic challenges.

### Inclusion and exclusion criteria

The inclusion criteria prioritized studies that correlated preoperative imaging findings with histopathological confirmation of GOC. Additionally, studies that analyzed the radiographic characteristics of GOC using CBCT, CT, MRI, or panoramic radiography were included. Case reports and retrospective studies that addressed diagnostic challenges and differential diagnoses based on imaging findings were also considered. Conversely, studies that focused exclusively on histopathological features without radiological correlation were excluded from the analysis. Additionally, review articles were omitted unless they were systematic reviews synthesizing imaging findings across multiple studies, ensuring that only original research and clinically relevant reports were included.

### Study selection process

Through a comprehensive search, 153 studies on GOC were initially identified. After applying predefined inclusion and exclusion criteria, 62 studies [8, 11–71] with relevant radiographic and histopathological data were selected for detailed analysis. From these, a total of 74 histopathologically confirmed cases of GOC were identified, all of which included preoperative imaging findings and differential diagnostic considerations prior to histopathological confirmation. These cases were systematically analyzed to assess common diagnostic challenges, imaging features, and differential diagnoses associated with GOC. Preoperative diagnostic terms and imaging characteristics reported in the literature were compiled and categorized to provide a structured overview of the most frequently observed radiological patterns and common misdiagnoses.

## Results

### Case series analysis of ten GOC cases

The ten histopathologically confirmed cases of GOC included in this study are summarized in Table 1. Patient age ranged from 31 to 69 years, with a mean of 49.1 (± 12.7) years. A male predominance was observed, with a male-to-female ratio of 7:3.

**Table 1:** Overview of the Ten GOC Cases Included in This Study

**Case**	**Age** **/Sex**	**Site**	**Clinical Finding **	**Radiographic finding **	**Preop. Dx.**	**Tx.**	**Bx.**	**F/U**	**Recur.**	Clinical progression*
1	61/M	Lt. Mn. body	Asymptomatic	Well-defined radiolucent lx.with slight buccolingual cortical thinning & expansion	OKC	SC	GOC	3m	N	12 yr interval; size ↑ >2×
2	42/M	Lt. Mn. body	Pain	moderately defined radiolucent lx. with scalloped border	OKC, AB	SC	GOC	65m	Y (55m)	3 yr interval; size ↑ >1.5×
3	55/M	Lt. Mn. ant.-body	Swelling, Pain	well-defined radiolucent lx. with scalloped border with buccolingual cortical thinning & expansion	OKC	Mar, SC	GOC	28m	N	–
4	42/M	Rt. Mn. body	Asymptomatic	well-defined radiolucent lx. (#44 apical area) with slight buccolingual thinning & expansion	RC (#44)	En	GOC	3m	N	–
5	31/F	Lt. Mn. body	Asymptomatic	moderately defined mixed lx.with slight buccolingual thinning & expansion	RC (#36)	En	GOC	4m	N	–
6	69/M	Lt. Mn. body	Swelling, Pain	moderately defined radiolucent lx.(#36area) with buccolingual thinning	RC (#36)	En	GOC	4m	N	–
7	46/F	Rt. Mn. body	Discomfort	moderately defined radiolucent lx. (#45-46 area)	RC (#45)	En	GOC	3m	N	–
8	58/M	Mn. ant.-Lt. Mn. body	Discomfort	moderately defined radiolucent lx. (#35-42) with buccally scalloped border	RC (#33), OKC	En	GOC	2m	N	–
9	58/M	Rt. Mn. body	Pain	well-defined & multilocular radiolucent lx. with buccolingual cortical thinning & expansion	OKC, AB	En	GOC	F/u lost	–	–
10	35/F	Rt. Mx. bnt.	Pain	moderately defined scalloped radiolucent lx. with slight labiopalatal cortical thinning	OKC	SC	GOC	1m	N	12 yr interval; size ↑ >2×

*GOC*, glandular odontogenic cyst; *OKC*, odontogenic keratocyst; *AB*, ameloblastoma; *RC,* radicular cyst; M; male; *F,* female; Lt.; left; Rt.; right; Mn.; mandible; Mx.; maxilla; ant.; anterior; lx.; lesion; Bx; biopsy; F/U; follow-up; Recur.; recurrence; m; month; *Y*, yes; *N*, no; Preop. Dx.; preoperative differential diagnosis; *Tx*, treatment; *SC*, surgical curettage; Mar.; marsupialization; *En*, enucleation; *yr*, year. Clinical progression* indicates the period between the initial diagnosis and first surgery; “interval” denotes the time elapsed and “size ↑” indicates lesion enlargement prior to treatment, not recurrence

The mandible was the most frequently affected site, accounting for 90% (9/10) of cases. Of these, seven were localized to the mandibular body, and two extended from the anterior region to the body. The sole maxillary case (10%) involved the anterior region. Seven patients presented with clinical symptoms, including pain alone (*n* = 3), swelling with pain (*n* = 2), and discomfort (*n* = 2).

**Fig. 1:**
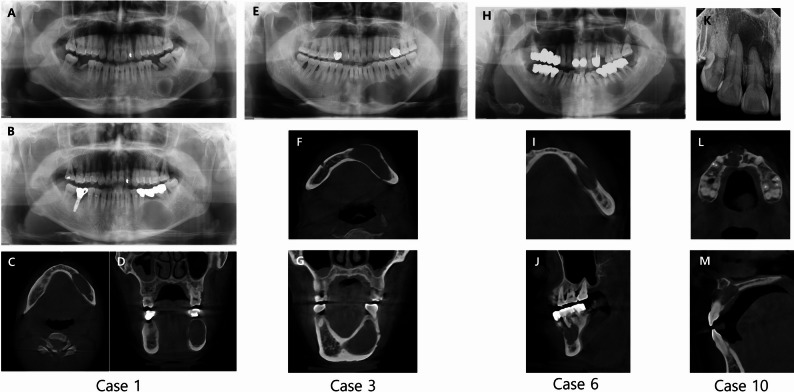
Case 1(**A** ~ **D**): **A **A Panoramic radiograph showed a well-defined, round-shaped unilocular lesion was observed in the left posterior mandible. **B **A follow-up panoramic image taken 12 years later showed that the lesion had more than doubled in size. **C**, **D **CBCT revealed buccolingual cortical thinning and expansion. Case 3(**E** ~ **G**): **E **A Panoramic image revealed well-defined radiolucent lesion with a scalloped margin was observed in the left mandibular anterior to posterior region. **F**, **G** CBCT revealed buccolingual cortical thinning and expansion. Case 6(**H** ~ **J**): A moderately defined radiolucent lesion was observed in the apical area of teeth #35 and #36, which had previously undergone endodontic treatment. Case 10(**K** ~ **M**): A moderately defined scalloped radiolucent lesion with slight labiopalatal cortical thinning was observed in the maxillary anterior region

**Fig. 2:**
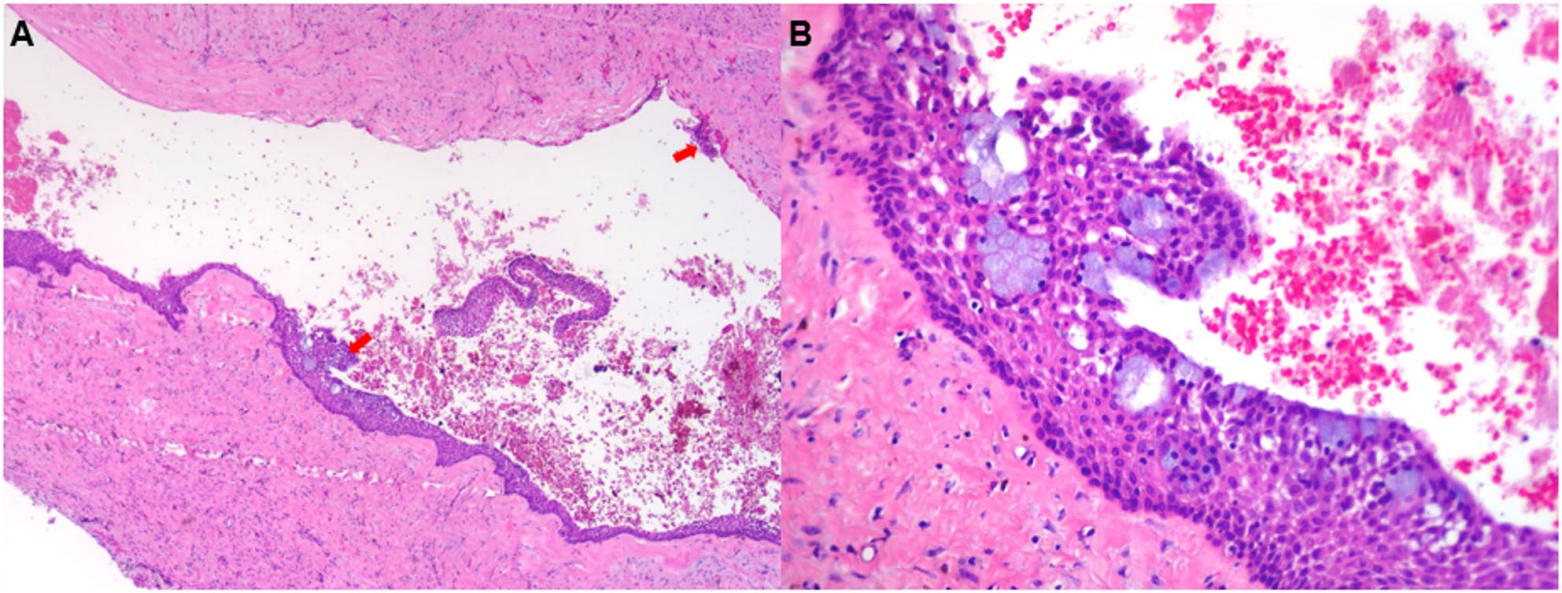
**A**: Low-power view of the Case 1 lesion demonstrates a lumen, cystic lining, and fibrous wall. The thickness of the epithelium varies, with some showing plaquelike thickening (red arrows). (hematoxylin and eosin [H&E] stain, ×40) **B** : The lining on the lumen side is cuboid or columnar in shape, has some cilia. Some microcysts and clusters of mucous goblet cells also observed within the lining epithelium in Case 1. (H&E stain x 200)

Radiographic analysis demonstrated marked variability in lesion characteristics, highlighting the diagnostic challenges associated with GOC. Lesion margins were classified as well-defined in 40% of cases and moderately defined in 60%. Most lesions (90%) were unilocular, with only one case exhibiting a multilocular pattern. Scalloped borders were present in 50% of cases, particularly in those initially suspected to be OKC. Among six cases preoperatively diagnosed as OKC, five showed scalloped margins. Cortical bone involvement was also variable: cortical thinning with expansion was noted in 60% of cases, while the remaining 40% showed thinning without expansion. Tooth involvement was observed in five cases (50%), all of which were misdiagnosed preoperatively as radicular cyst (RC). However, none of these cases exhibited root resorption, tooth displacement, or association with impacted teeth—features commonly observed in other odontogenic cysts. The primary preoperative differential diagnoses included OKC (*n* = 5) in five cases and RC (*n* = 5). In addition, AB was listed as a secondary consideration in two cases, and OKC in one other case, reflecting diagnostic overlap in several patients. Figure 1 illustrates four representative cases of GOC that were initially interpreted as OKC or RC based on radiographic features. Figure 2 illustrates the characteristic histopathological features of GOC, as observed in a representative case.

In three cases, the lesion size increased by approximately 1.5- to 2-fold during the interval between the initial diagnosis and the first surgery. All patients underwent surgical curettage or enucleation. One patient (case 3) required marsupialization prior to definitive surgery due to the lesion’s large size. Recurrence (case 2) was observed in a single case (10%) after 55 months, while the remaining nine patients showed no evidence of recurrence during follow-up periods ranging from 1 to 65 months.

### Literature review

The summarized demographic and clinical features of 74 reported GOC cases are presented in Table 2.

**Table 2:** Summary of 74 Reported Cases of Glandular Odontogenic Cyst: A Literature Review

Age (*n*=74)	
Mean ± SD: 44.37 ± 18.53 years	
Sex (*n*=74)	
Male 39 (52.7%)	
Female 35 (47.3%)	
Anatomical Location (*n*=72)	
Maxilla 18 (25%)	
Anterior region 10 (13.9%)	
Posterior region 3 (4.2%)	
Anterior-posterior region 4 (5.6%)	
Maxillary sinus region 1 (1.4%)	
Mandible 54 (75%)	
Anterior region 22 (30.6%)	
Posterior region 22 (30.6%)	
Anterior-posterior region 9 (12.5%)	
Ramal region 1 (1.4%)	
Size (*n*=26)	
Mean ± SD: 3.15 ± 1.97 cm	
Symptoms (*n*=69)	
Asymptomatic 12 (17.4%)	
Symptomatic 57 (82.6%)	
Swelling 40 (58.0 %)	
Swelling and pain 8 (11.6%)	
Pain 4 (5.8%)	
Pressure 2 (2.9%)	
Pressure and pain 1 (1.4%)	
Others 2 (2.8 %) (pus discharge 1, tooth mobility 1)	
Treatment (*n*=69)	
Enucleation 40 (58.0%)	
Mandibular resection 17 (24.6%)	
Curettage 4 (5.8%)	
Marsupialization 3 (4.4%)	
Others 5 (7.3%) [incisional biopsy 2, surgical exploration 2, cystectomy 1]	
Follow-up (*n*=48)	
Mean ± SD: 25.27 ± 26.20 months	
Recurrence (*n*=48)	
Yes 7 (14.6%)	
No 41 (85.4%)	
Mean ± SD: 30.14 ± 33.44 months	

*SD*; standard deviation

### Age and sex distribution of GOC cases

The mean age of the patients was 44.37 years (± 18.53), reflecting the broad age distribution of individuals affected by GOC. A slight male predominance was observed, with 39 cases (52.7%) occurring in males and 35 cases (47.3%) in females.

### Anatomical distribution and lesion size

GOC predominantly occurs in the mandible, accounting for 75% of cases, while the maxilla is affected in 25% of cases. Within the maxilla, the anterior region is the most commonly involved site (10 of 18 cases; 55.6%), whereas in the mandible, the anterior and posterior regions are equally affected, each comprising 22 of 54 cases (40.7%). Lesions extending across the anterior and posterior regions were observed in 16.7% of mandibular cases (9 of 54) and 22.2% of maxillary cases (4 of 18), indicating a slightly higher tendency for extensive involvement in the maxilla. Additionally, involvement of the ramus (1 of 72) and maxillary sinus (1 of 72) was rare, occurring in approximately 3% of cases. The mean lesion size was 3.15 ± 1.97 cm. 

### Symptoms

Among the 69 patients with available clinical data, 57 cases (82.6%) were symptomatic, while 12 cases (17.4 %) were asymptomatic. Swelling was the most frequently reported symptom, occurring in 40 cases (58.0%), followed by swelling accompanied by pain (8 cases, 11.6%), and pain as an isolated symptom (4 cases, 5.8%). Pressure-related symptoms were less common, with 2 cases (2.9%) reporting pressure sensation and 1 case (1.4%) presenting with both pressure and pain. Additionally, rare findings such as pus discharge and tooth mobility were each observed in 1 case (1.4%), totaling 2.8% overall.

### Treatment and recurrence

Treatment data were available for 69 cases, with the most commonly performed procedure being enucleation (40 cases, 58%). Other treatment approaches included mandibular resection (17 cases, 24.6%), curettage (4 cases, 5.8%), and marsupialization (3 cases, 4.4%). Additionally, five cases (7.3%) underwent alternative procedures, including incisional biopsy (2 cases), surgical exploration (2 cases), and cystectomy (1 case). Follow-up data were available for 48 patients, with a mean follow-up duration of 25.27 ± 26.20 months. Recurrence was observed in 7 cases (14.6%), whereas 41 cases (85.4%) showed no evidence of recurrence. The mean interval to recurrence was 30.14 ± 33.44 months, indicating variability in the timing of recurrence among affected cases.

### Radiological characteristics

The detailed radiographic characteristics of 74 reported GOC cases are summarized in Table 3. Radiographic analysis of 74 cases of GOC demonstrated that 72 cases (97.3%) presented as radiolucent, while 2 cases (2.7%) exhibited mixed radiolucent-radiopaque features. Among 58 cases with documented locularity, 28 cases (48.3%) were unilocular, and 30 cases (51.7%) were multilocular. The lesion margins were predominantly well-defined (41 of 44 cases; 93.2%), with scalloped borders observed in 8 of 44 cases (18.2%). Cortical bone involvement was noted in 22 cases, including cortical thinning (9 cases, 40.9%), cortical expansion (9 cases, 40.9%), and cortical perforation (4 cases, 18.2%). Tooth-related changes were reported in 36 cases, with tooth displacement (14 cases, 38.9%) being the most common finding, followed by root resorption (6 cases, 16.7%), impacted tooth association (5 cases, 13.9%), edentulous area involvement (4 cases, 11.1%), association with a missing tooth (4 cases, 11.1%), and association with a non-vital tooth (3 cases, 8.3%).

**Table 3:** Radiographic Characteristics of 74 Reported Cases of Glandular Odontogenic Cyst: A Literature Review

Radiolucency (*n*=74)	
Radiolucent 72 (97.3%)	
Mixed 2 (2.7%)	
Locularity (*n*=58)	
Unilocular 28 (48.3%)	
Multilocular 30 (51.7%)	
Margin (*n*=44)	
Well-defined 41(93.1%)	
Scalloped border 8 (18.2%)	
Ill-defined 3 (6.8%)	
Cortical bone involvement (*n*=22)	
Cortical thinning 9 (40.9%)	
Cortical expansion 9 (40.9%)	
Perforation 4 (18.2%)	
Tooth involvement (*n*= 36)	
Tooth displacement 14 (38.9%)	
Root resorption 6 (16.7%)	
Involvement of impacted tooth 5 (13.9%)	
Association with missing tooth 4 (11.1%)	
Association with edentulous region 4 (11.1%)	
Association with non-vital tooth 3 (8.3%)	

### Preoperative differential diagnosis

The distribution of preoperative and comprehensive differential diagnoses of GOC cases is summarized in Table 4. Among the 74 confirmed cases of GOC, 132 preoperative diagnoses were recorded, averaging 1.78 per case and reflecting the diagnostic complexity of the lesion. The most frequently suspected conditions were AB (24.3%) and OKC (21.6%) as primary diagnoses, with their total mention rates rising to 24.2% and 25.0%, respectively—collectively accounting for nearly half of all preoperative assessments. RCs (13.5%) and dentigerous cysts (12.2%) were also common misdiagnoses, each appearing in 9.1% of total differential considerations. Other odontogenic benign tumor or cyst such as calcifying epithelial odontogenic tumor (CEOT), adenomatoid odontogenic tumor (AOT), central giant cell granuloma (CGCG), odontoma, lateral periodontal cyst, and BOC comprised 9.5% of primary diagnoses and 12.1% of total mentions. Residual cysts (8.1%) and mucinous epidermoid carcinoma (4.1%) were occasionally considered, though less frequently mentioned overall (4.6% and 2.3%, respectively). Notably, inflammatory and non-odontogenic lesions such as chronic apical periodontitis and adenoid cystic carcinoma (ACC) were classified under “Others,” comprising only 2.7% of primary diagnoses but 8.3% of total mentions, underscoring the broad diagnostic spectrum considered prior to histopathologic confirmation. Odontogenic myxoma and fibro-osseous lesions were rarely included in differential considerations, accounting for only 1 to 2 of primary diagnoses (≤3%).

**Table 4:** Comparison of preoperative radiological diagnostic considerations in glandular odontogenic cyst: primary vs. Comprehensive differential diagnoses (Literature review of 74 cases)

Radiological Differential Diagnosis	Primary Radiological Impression, *n* (%)	Overall Differential Consideration, *n* (%)
Odontogenic keratocyst Ameloblastoma Radicular cyst Dentigerous cyst *Benign tumor or cyst Residual cyst Mucoepidermoid carcinoma Odontogenic myxoma **Fibro-osseous lesion ***others	16 (21.6) 18 (24.3) 10 (13.5) 9 (12.2) 7(9.5) 6 (8.1) 3(4.1) 1(1.4) 2(2.7) 2 (2.7)	33 (25.0) 32(24.2) 12(9.1) 12(9.1) 16(12.1) 6(4.6) 3(2.3) 5(3.8) 2(1.5) 11(8.3)
Total	74 (100)	132 (100)

*Benign tumor or odontogenic cyst: *CEOT*, *AOT*, *CGCG*, Odontoma, Lateral periodontal cyst, *BOC*, **Fibro-osseous lesion; ossifying fibroma or simple bone cyst, ***Others: chronic apical periodontitis, ACC

## Discussion

Traditionally, GOC has been considered a rare lesion primarily affecting the anterior mandible, with small, unilocular radiolucencies [4, 6]. However, our findings challenge this notion. Nine out of ten institutional cases occurred in the posterior mandible, with many demonstrating large or multilocular features that radiographically resembled OKC or RC. A literature review confirmed similar trends, with a predominance of mandibular involvement and frequent misdiagnoses as OKC or AB. These findings suggest that GOCs can present more diversely than previously recognized and should be considered in the differential diagnosis of large, multilocular mandibular lesions.

While OKC was the most frequent preoperative misdiagnosis in both institutional and literature cases, a higher proportion of RC diagnoses were observed in our institutional data. This may be due to unilocular appearance, periapical location, or clinical assumptions based on tooth vitality. Literature-based cases more frequently referenced AB, possibly reflecting publication bias toward more aggressive presentations. These discrepancies highlight how lesion location, size, and clinical context influence diagnostic impression.

Our case series included more asymptomatic patients (30.0%) than literature cases (17.4%), indicating that GOCs may be incidentally discovered on imaging. Radiographically, our cases were predominantly unilocular, contrasting with the near-equal distribution of unilocular and multilocular lesions reported in the literature. Only one case in our series exhibited multilocularity. This discrepancy may reflect earlier detection or sampling variability. Yeom et al. emphasized that many jaw lesions, including cystic pathologies, are frequently asymptomatic and discovered incidentally on panoramic radiographs, highlighting the need for vigilant radiographic interpretation and standardized diagnostic protocols [72]. In this context, misdiagnosis or delayed identification of GOC is not uncommon, particularly in cases lacking overt clinical symptoms.

Additionally, this study provides new insights into the growth pattern of GOCs. While GOCs have been described as potentially aggressive, our findings indicate that not all cases exhibit rapid enlargement. In the 10 case reports analyzed, only three showed a significant increase in lesion size, ranging from 1.5 to 2 times larger over time. This contrasts with the assumption that GOCs consistently exhibit aggressive growth and suggests that the rate of enlargement varies significantly between cases. Furthermore, size variability reported in the literature (mean lesion size of 3.15 ± 1.97 cm) supports the notion that GOCs do not follow a uniform growth trajectory and may remain stable for extended periods before showing notable expansion.

Radiographic analysis of the ten institutional cases revealed notable differences from previous literature. While prior studies have reported a nearly equal distribution of unilocular (48.3%) and multilocular (51.7%) GOCs, most cases in this study presented as well-defined unilocular lesions, with only one case exhibiting a multilocular appearance. This discrepancy may be related to differences in sample size, lesion progression, or earlier detection. Preoperative misdiagnosis patterns were consistent with prior reports, where OKC (25.0%) and AB (24.2%) were the most frequently suspected lesions [6, 8]. In our series, OKC (6 of 10 cases), RC (5 of 10 cases), and AB (2 of 10 cases) were the most common preliminary diagnoses, reflecting the ongoing diagnostic challenge due to overlapping radiographic features. Interestingly, 50% of our cases were initially misdiagnosed as RC—a markedly higher rate than the 13.5% reported in the literature [73]. This suggests that GOCs near tooth apices may be more frequently mistaken for inflammatory cysts than previously recognized. Radiographically, GOC can resemble RC, presenting as periapical radiolucencies [74]; however, they differ in origin and behavior. This supports the necessity of histopathological confirmation to avoid misdiagnosis. Moreover, although GOCs can radiographically mimic OKC or AB, subtle differences exist. OKCs often show more prominent internal septations and buccolingual expansion, whereas ABs present with coarser septa and aggressive bone destruction [6, 75]. In contrast, GOCs tend to have a more uniform internal structure with less cortical expansion. Reviews by Fowler et al. and MacDonald-Jankowski have highlighted these diagnostic complexities and emphasized the importance of recognizing diverse radiological patterns in GOCs [6, 8].

Advanced imaging modalities such as CBCT and MRI have been explored for GOC diagnosis, but neither provides definitive criteria, necessitating histopathological confirmation [10, 76]. CBCT effectively assesses cortical bone involvement, perforation, and expansion, with studies reporting cortical plate loss in 71% and significant bone expansion in 62% of cases [10]. It also aids in surgical planning by detailing buccolingual expansion but lacks specificity in soft tissue differentiation [76]. MRI offers superior soft tissue contrast, distinguishing solid and cystic components, particularly in GOC-associated AB [77]. However, its enhancement patterns overlap with dentigerous cysts, RC, and central MEC, limiting its diagnostic reliability [77]. These results reinforce the need for a multimodal approach integrating imaging with histopathology for accurate diagnosis and treatment planning [76].

GOC is considered to originate from residual dental lamina, supporting its odontogenic nature and explaining its predilection for tooth-bearing areas and its overlap with other odontogenic cysts and tumors [5, 6, 10]. Histopathologically, GOC exhibits a broad spectrum that often overlaps with central MEC and other cysts such as the lateral periodontal cyst [6, 75]. Key distinguishing features include mucin-filled cystic spaces, gland-like or duct-like structures, intraepithelial microcysts, variable epithelial thickness with eosinophilic cuboidal (hobnail) cells, and the absence of infiltrative epithelial proliferation or cytologic atypia [6, 11, 68, 75]. According to the 5th edition of the WHO Classification (2022), these features remain essential for diagnosis, whereas the presence of MAML2 gene rearrangement is regarded as a molecular hallmark of central MEC [5]. Molecular studies have shown that GOC typically lacks MAML2 gene rearrangements, reinforcing its odontogenic rather than salivary origin and confirming that it is distinct from MEC [6, 10]. In the present series, additional molecular testing could not be performed due to the retrospective nature of the study; however, all ten cases fulfilled the Fowler et al. (2011) histopathologic criteria for GOC, and nine of them occurred in the posterior mandible, showing well-defined corticated borders without a solid infiltrative component—findings that favor GOC over central MEC [6, 10, 75]. Given the histologic and radiologic overlap between these entities, a multidisciplinary approach integrating clinicopathologic assessment, advanced imaging, and, when feasible, molecular analysis (e.g., MAML2 rearrangement testing) is recommended to ensure accurate diagnosis and appropriate management [5, 10, 51].

Given the diagnostic ambiguity of GOC on conventional radiographs, future studies should prioritize the integration of artificial intelligence (AI)-driven diagnostic tools to support differential diagnosis. Recent developments in deep learning algorithms have demonstrated promising results in differentiating morphologically similar jaw lesions using panoramic radiographs [78]. For instance, Lee et al. developed a DenseNet-based model that successfully distinguished Stafne’s bone cavity from various odontogenic cysts and tumors with over 99% accuracy, despite variability in imaging systems and lesion appearance [79]. This suggests that deep learning frameworks can effectively recognize subtle radiographic cues that may elude human observers, offering a valuable adjunct in distinguishing GOC from similar-appearing lesions such as OKC or AB on panoramic and CBCT images. Moreover, radiomics-based analysis utilizing CBCT has shown high efficacy in discriminating lesion subtypes. Sha et al. reported that texture-based radiomic signatures derived from CBCT could differentiate between conventional and AB, demonstrating the potential of this approach for nuanced classification tasks [80]. In parallel, Cai et al. illustrated the use of digital pathology-based AI for lesion subclassification and prognosis, which could complement imaging data in establishing integrated diagnostic models [81]. Leveraging such models, future GOC studies should aim to construct a multimodal diagnostic pipeline that fuses radiographic, radiomic, and possibly histopathologic features into a unified AI-assisted diagnostic framework, thereby improving early detection, reducing misdiagnosis, and guiding treatment planning with greater confidence.

This study demonstrates the diagnostic challenges of GOC and underscores the necessity of histopathologic confirmation due to its overlapping radiographic features with other odontogenic lesions. However, several limitations should be acknowledged. This single-institution retrospective study, comprising 10 cases, is underpowered for risk modeling and inherently subject to selection and information bias. Moreover, molecular testing (e.g., *MAML2* rearrangement) was not performed, limiting the exclusion of intraosseous MEC in histologically borderline lesions. Another drawback is the limited availability of long-term postoperative follow-up data, despite the relatively high recurrence rate of GOC. Given the potential for late recurrence, future studies should incorporate extended observation periods to better assess treatment outcomes and recurrence patterns. Additionally, the small sample size limits generalizability, as GOC characteristics may vary across geographic, ethnic, and institutional settings. Prospective multi-center studies with standardized imaging protocols and ancillary molecular testing are needed to refine radiologic predictors, validate diagnostic criteria, and optimize management algorithms.

## Conclusion

This study challenges the traditional view that GOC is small, unilocular lesions confined to the anterior mandible. The differing patterns of misdiagnosis in our cases and the literature—ranging from RC to AB—underscore the wide radiographic spectrum of GOC. This variability underscores the diagnostic ambiguity of GOC and highlights the importance of considering them in the differential diagnosis of large, multilocular jaw lesions. A multidisciplinary diagnostic approach, incorporating clinical, radiologic, and histopathologic evaluation, is essential for accurate diagnosis. Future integration of artificial intelligence and radiomics may further enhance diagnostic precision and improve clinical outcomes.

## Data Availability

All data analyzed during this study are included in this published article.

## Abbreviations

GOC: Glandular odontogenic cyst
CBCT: Cone-beam computed tomography
OKC: Odontogenic keratocyst
RC: Radicular cyst
AB: Ameloblastoma
AI: Artificial Intelligence
WHO: World Health Organization
MEC: mucoepidermoid carcinoma
MeSH: Medical Subject Headings
MRI: Magnetic resonance imaging
CEOT: Calcifying epithelial odontogenic tumor
AOT: Adenomatoid odontogenic tumor
CGCG: Central giant cell granuloma
BOC: Botryoid odontogenic cyst
ACC: Adenoid cystic carcinoma
